# Supplementary material for: A genomic basis of vocal rhythm in birds
Source: Nat Commun. 2024 Apr 23;15:3095. doi: 10.1038/s41467-024-47305-5 (PMC11039653; doi:10.1038/s41467-024-47305-5)
Supplement: Supplementary file 1 — Supplementary Information [file 41467_2024_47305_MOESM1_ESM.pdf]

Supplementary Information

A genomic basis of vocal rhythm in birds

Matteo Sebastianelli[1, 2*], Sifiso M. Lukhele[1], Simona Secomandi[1], Stacey G. de Souza[1], Bettina Haase[3], Michaella Moysi[1], Christos Nikiforou[1], Alexander Hutfluss[4], Jacquelyn Mountcastle[3], Jennifer Balacco[3], Sarah Pelan[5], William Chow[5], Olivier Fedrigo[3], Colleen T. Downs[6], Ara Monadjem[7,8], Niels J. Dingemanse[4], Erich D. Jarvis[3,9,10], Alan Brelsford[11], Bridgett M. vonHoldt[12], and Alexander N. G. Kirschel[1*]

Corresponding author: matteo.sebastianelli@imbim.uu.se, kirschel@ucy.ac.cy

Table of contents:

- Supplementary Methods
    - Supplementary Methods 1 - Sample collection and storage
    - Supplementary Methods 2 - Genomic data extraction, library preparation and sequencing
    - Supplementary Methods 3 - Chromosome-level reference genome assembly and evaluation
    - Supplementary Methods 4 - Whole-genome variant calling
    - Supplementary Methods 5 - Double-digest restriction site associated DNA sequencing (ddRAD)
    - Supplementary Methods 6 - Identifying candidate loci underlying IOI
    - Supplementary Methods 7 - Estimating timing of admixture in the contact zone
    - Supplementary Methods 8 - Hybrid song instability models and post hoc test
    - Supplementary Methods 9 - Quantification of playback effects
    - Supplementary Methods 10 - Estimation of repeatability
    - Supplementary Methods 11 - Assessing covariance of phenotypes
- Supplementary Figures S1 to S15
- Supplementary Tables S1 to S15
- References

Supplementary Methods

Supplementary Methods 1 - Sample collection and storage

Blood samples were obtained from each banded individual through venipuncture of the brachial vein and stored in 1ml queen's lysis buffer (aliquoted from 800 ml dH2O; 1,22g Tns-Cl; 0,6 NaCl; 200 ml EDTA; 10g n-lauroylsarcosine; pH 8) for 443 samples or 100% ethanol (1ml $\times$ 9 samples). This total included 71 samples obtained for a previous study[1]. Two samples of *pusillus* were collected with the aim of assembling a reference genome at Mlawula Game Reserve, Eswatini in July 2019, with blood stored in 100% ethanol and placed on dry ice in the field, before transfer to a -80 °C freezer prior to shipping to the Vertebrate Genomes Lab, at the Rockefeller University for sequencing.

We obtained spectral measurements of the plucked forecrown feathers using a JAZ spectrometer (Ocean Optics) attached to a fiber-optic reflectance probe (Ocean Optics R-200) and PX xenon light source to obtain two spectra measurements: one parallel and one perpendicular orientation measurement from each of 87 ringed-recorded individuals. Each reflectance spectrum was measured relative to the reflectance of WS-2 white standard (Ocean Optics) and a black standard by blocking the light path of the spectrometer by replacing its cap. Replicate spectra were averaged, processed and hue H4 values computed in the R package pavo v2.9.0[2].

Supplementary Methods 2 - Genomic data extraction, library preparation and sequencing

DNA extraction was performed using a modified protocol of the Bionano Prep SP Frozen Human Blood DNA Isolation Protocol for the Bionano Prep SP Blood and Cell Culture DNA Isolation Kit (cat no. 80030). Briefly, the input volume was adjusted to 15 µl of blood and then brought to a total of 40 µl with chilled 1x PBS. The lysis and digestion were performed on the whole blood and PBS mixture. Isolated high molecular weight DNA was kept at room temperature for a week to homogenize before quantified with a Qubit 3 fluorometer (Invitrogen Qubit dsDNA Broad Range Assay cat no. Q32850). DNA fragment size distribution was assessed using a pulsed field gel electrophoresis (PFGE; Pippin Pulse, SAGE Science, Beverly, MA) and found the majority of DNA was over 300 Kb. The obtained DNA was then used to prepare the libraries for the different sequencing technologies. For PacBio libraries, DNA was sheared down to ~40 Kb fragment size by needle shearing. To prepare PacBio large insert libraries, the SMRTbell express template pre kit 2.0 (no. 100-938-900) was used. Using Sage BluePippin (Sage Science, USA), libraries were size-selected for 15 Kb, and were then sequenced on three 8M SMRT cells with the Sequel II instrument, using the Sequel II sequencing kit 2.0 (no. 101-820-200) and 15-h movie time.

To generate linked reads, the extracted DNA was processed on the 10X Genomics Chromium platform (Genome Library Kit & Gel Bead Kit v2 PN-120258, Genome Chip Kit v2 PN-120257, i7 Multiplex Kit PN-120262) following manufacturer's guidelines. The libraries were then sequenced on an Illumina NovaSeq 6000 S4 150-bp PE lane at ~60× coverage. For Bionano libraries, the DNA was labeled using direct labeling enzyme (DLE1) following the DLS protocols (document number 30206). The samples were then imaged on a Bionano on a Bionano Saphyr instrument. Finally, chromatin interaction (Hi-C) libraries were generated by Arima Genomics (https://arimagenomics.com/) from the blood samples with in vivo cross-linking using the two-enzymes Arima-HiC kit (P/N: A510008). The proximally ligated DNA was sheared, size-selected around 200-600 bp with SPRI beads, and enriched for biotin-labeled DNA using streptavidin beads. Illumina libraries were then generated from the fragments using KAPA Hyper Prep kit (P/N: KK8504) and subsequently amplified through PCR and purified using SPRI beads. After a quality check with qPCR and Bioanalyzer, the libraries were sequenced on Illumina HiSeq X at ~60× coverage following the manufacturer’s protocols.

For population genomics analyses, we sequenced 137 further tinkerbirds at the whole-genome level (Supplementary Data File 1) on Illumina NovaSeq 6000 S4 150-bp PE lanes (median depth = 8.53X; mean depth = 8.21X), of which 52 were assigned to *extoni* and 85 *pusillus* (including 28 allopatric *extoni* and 12 allopatric *pusillus*) based on forecrown color. We also sequenced a total of 452 tinkerbird samples, including 123 of those sequenced with WGS, using double-digest restriction site associated DNA sequencing (ddRAD), 71 of which were included in a previous study[1] (Supplementary Data File 2).

From each sample, 0.4 μg of DNA was used for whole-genome sequencing. Libraries were generated using the NEBNext DNA Library Prep Kit following the manufacturer's recommendations. The PCR products were then purified using the AMPure XP system, analyzed for size distribution (by Agilent 2100 Bioanalyzer) and quantified using real-time PCR. The libraries obtained with this method were then sequenced with the Illumina Novaseq6000 platform.

The ddRAD sequencing procedure also involved multiple stages. The first stage includes digestion of DNA with restriction enzymes (*Sbf*I and *Mse*I). We prepared a first master mix with the restriction enzymes and then combined the master mix with individual DNA samples in each plate well. The combination master mix with DNA was then vortexed, centrifuged and incubated. The following stage was the ligation of barcoded adapters which included thawing the adaptors, preparation of a second master mix, the addition of this master mix to each well of the restriction digested DNA and finally the addition of a unique barcoded adaptor for each DNA sample. The third stage involved purification (removal of short fragments) using Agencourt AMPure. AMPure purification consists of: i) addition of AMPure beads to the DNA volume, ii) binding, iii) separation of the beads from the solution by placing the reaction plate onto a magnetic plate, iv) ethanol wash by adding a specific amount of ethanol to each well of the plate, v) addition of the elution buffer and vi) transfer of the eluent with DNA into a new plate. Subsequently, the next stage includes the amplification of each individual sample in four separate PCR reactions. This PCR step used the Illumina PCR primers to amplify fragments that have our adapters and barcodes ligated onto the ends. To improve stochastic differences in PCR production of fragments in reactions, we ran four separate reactions per restriction-ligation product to then combine them. We prepared a third master mix with standard PCR reagents including Q5 high-fidelity polymerase, and added the combined master mix III to each plate well

followed by the addition of the diluted restriction-ligation purified with AMPure mix. Next, we ran the PCR for 20 thermal cycles. Subsequently, to maximise the amount of double-stranded DNA and reduce DNA heterodimer content, we prepared another master mix (master mix IV) with additional PCR primers and dNTPs, added it to each PCR product and then ran one additional thermal cycle. In the following phase, we evaporated the PCR product to about ½ volume and pooled the PCR product from the four replicates and all samples into one tube. Then we ran the PCR product on an agarose gel and selected fragments between 400 and 500 bp in order to exclude fragments that consist mostly of adaptor sequence. We then conducted a final AMPure cleanup on the pooled library and shipped it to Novogene Inc. for 150 bp paired-end sequencing on an Illumina HiSeq X Ten platform.

Supplementary Methods 3 - Chromosome-level reference genome assembly and evaluation

PacBio subreads were assembled into contigs with FALCON[3] v. 1.3.0 and haplotypes further phased with FALCON-Unzip[4] v. 1.2.0. A set of primary contigs, representing the principal pseudo-haplotype, and a set of alternate haplotigs, representing the alternate haplotype, were generated. Purge_dups[5] was used on the primary contigs to remove any retained alternate haplotigs, overlaps, collapsed repeats and low- and high-coverage contigs. The alternate haplotigs were merged together in a single file and purged again, while the primary purged contigs were subjected to three steps of scaffolding.

The first scaffolding step was performed with Scaff10X v2.0-2.1 (https://github.com/wtsi-hpag/Scaff10X) using 10x linked reads to join proximal contigs into larger scaffolds. The resulting set of scaffolds were then scaffolded with Bionano DLS optical maps[6] using Bionano Solve v3.2.1 in non-haplotype assembly mode with a DLE-1 one enzyme non-nicking approach. The third and last step of scaffolding was performed aligning the Hi-C reads to the scaffold assembly using the Arima Genomics mapping pipeline (https://github.com/ArimaGenomics/mapping_pipeline) and scaffolded using Salsa2 HiC v. 2.2[7].

To improve the assembly per-base accuracy (QV)[8], the primary scaffolded assembly was merged with the alternate combined haplotigs and the mitogenome to prevent switches and NUMTs overpolishing[8,9], and three steps of polishing were performed. The first step of polishing was with Arrow v. SMRTLink7.0.1 (PacificBiosciences) using PacBio CLR reads. The last two rounds of polishing were done with Longranges Align v. 2.2.2 and freebayes[10] v. 1.3.1 using 10x

linked reads. Primary scaffolded assembly and alternate haplotigs were then separated again and called with the prefix 'bPogPus1', on the basis of the VGP guidelines for genome identifiers[8]. Manual curation was then performed on the primary assembly to remove any remaining false duplications, correct structural assembly errors, and assign scaffolds to chromosome names. Curation also included a custom decontamination pipeline[11], the genome browser gEVAL[12] v. 2020-05-14, HiGlass Hi-C 2D maps and pretextView (https://github.com/wtsi-hpag/PretextView)[13] were used. The final curated assembly was evaluated with gfastats[14], Merqury[15] (21bp *k*-mer) and BUSCO[16] ('aves' BUSCO genes) on the European Galaxy Server[17]. Following Secomandi *et al.*, 2023 (https://github.com/SwallowGenomics/BarnSwallow/blob/main/Analyses/assembly_evaluation/assembly_evaluation.txt)[18], we masked the genome with a combination of Windowmasker v1.0.0[19] and RepeatMasker v4.1.4[20] using the RepBase v20,170,127 database[21].

Supplementary Methods 4 - Whole-genome variant calling

To process raw whole-genome sequences of 137 *extoni* and *pusillus* we first proceeded to trim Illumina adapter sequences using Cutadapt[22] v. 4.0 and merged pair-end reads to produce single interleaved FASTQ files. We then aligned the reads to the recently assembled reference genome of a female red-fronted tinkerbird (*pusillus*: NCBI BioProject: PRJNA637953) using BWA-mem[23] v. 0.7.1 , and included read group information. We also aligned the raw 10x linked reads of the reference genome individual to the reference for inclusion in subsequent analyses. Subsequently, we used SAMtools[24] v. 1.9 to filter out mapped reads with mapping quality below 20 and sort the data by coordinate before converting SAM to BAM files. We then removed duplicate reads with Picard's MarkDuplicates tool[25] (version 2.23.1). We performed all subsequent processes in GATK[26] v.4.0.11.0. We used HaplotypeCaller for individual-level variant calling, we then combined samples using CombineGVCFs and performed joint genotyping with GenotypeGVCFs. This process resulted in a single VCF file which we then filtered using GATK's recommended hard filtering parameters (QUAL < 30.0, QD < 2.0, SOR > 3.0, FS > 60.0, MQ < 40.0, MQRankSum < -12.5 and ReadPosRankSum < -8.0) to produce two variant files, one containing SNPs and the other containing indels. We then used these variants to implement base quality score recalibration (BQSR), in which the base quality scores of the BAM files, obtained after removing duplicate reads, were adjusted in a two-step process. Firstly, we

used BaseRecalibrator to build a recalibration model for each BAM file along with the variant files previously produced. Secondly, we used ApplyBQSR to adjust the base quality scores within each BAM based on the recalibration model, to produce a new recalibrated BAM file. Additionally, we built a second model from the recalibrated BAMs and produced 'before-and-after' plots to visualize the effects of the recalibration process using BaseRecalibrator and AnalyseBQSR, respectively. Using the recalibrated BAMs, we then called variants on an individual level, combined samples and performed joint genotyping, to produce the final VCF file, using the same tools described above. We finally applied filters to create two high-quality datasets for subsequent analyses that have different data requirements. Using VCFtools[27] v. 0.1.16, we first created a filtered dataset with the following parameters: maximum missingness = 0.95; minor allele frequency = 0.03; minimum depth = 3.5 and maximum depth = 50, and then created a second dataset with the same filter parameters excluding the minor allele frequency (hereafter 'no MAF' dataset). The no MAF dataset also contained invariant sites, which were necessary to calculate statistics such as π and D_{XY}, whereas we used the MAF filtered dataset in all the other analyses unless noted. Overall, from the initial pre-filtered vcf files of 65,812,270 and 631,918,066 SNPs (with invariant sites), the above-mentioned pipeline resulted in a final dataset of 19,602,343 SNPs for the MAF filtered dataset and 303,114,882 SNPs for the no MAF dataset. Upon further exploration of the whole-genome dataset, we removed two allopatric individuals of each species that we deemed had been mislabelled and another allopatric *extoni* which showed signs of contamination, possibly during DNA extraction, with the final dataset consisting of 135 individuals.

Supplementary Methods 5 - Double-digest restriction site associated DNA sequencing (ddRAD)

We demultiplexed the raw reads using process_radtags along with a list including barcodes associated with individual samples to produce single sample FASTQ files for each paired-end read. Subsequently, we removed PCR clones using clone_filter. We then mapped and aligned the reads to the *P. p. pusillus* reference genome (bPogPus1.pri.cur.20200514.fasta) using BWA-mem v. 0.7.1. Using SAMtools v. 1.9, we filtered out mapped reads with mapping qualities < 20 and sorted the reads by coordinate before converting from SAM to BAM. Following this, we called variants and genotyped using *gstacks* in Stacks (using --*var-alpha* 0.01 and --*gt-alpha* 0.01)[28].

Lastly, population genetics statistics were obtained using *populations* in Stacks together with a pop.map file specifying the population of each sample. Ultimately, we produced a VCF file with 82,950 SNPs after filtering out variants that had a depth < 4, > 20% missing genotype data, and a minor allele frequency < 5%, resulting in a median depth = 12.85X and a mean depth = 13.92X.

Supplementary Methods 6 - Identifying candidate loci underlying IOI

GEMMA LMM (*-lmm 1* flag) output was analyzed and interpreted based on significant SNPs that passed a significance threshold set by permutation, which was performed by running 999 LMMs in GEMMA after shuffling phenotypes at random in each run. We set two thresholds, with the most stringent being the mean value of the most significant SNP from each GEMMA run ($-log_{10} P$ = 6.9, rounded to 7) and the less stringent threshold established by averaging the p-value of the 10 most significant SNPs across each run ($-log_{10} P$ = 6.1, rounded to 6). We investigated just those clusters with at least three significant SNPs. Clusters of significant SNPs were then located in the *pusillus* reference genome after aligning them to the zebra finch (*Taenopygia guttata*) genome using BLAST[29]. We then performed the reverse process: after identifying potential genes that underlie the expression of IOI, we extracted their exact gene position from the annotated zebra finch genome and used BLAST to locate the genes in the tinkerbird genome. Following this, from the GEMMA output we extracted beta values and standard error only for the SNPs in the gene regions where the significant SNPs mapped into in order to calculate their effect size. We then compared the effect sizes of each gene against 10,000 SNPs randomly chosen across the entire genome. This was done by estimating the standard error over the mean (SEM), a measure of the variance explained by each gene.

In GEMMA, we also analysed the genetic architecture of IOI using Bayesian sparse linear mixed models (BSLMM), for which we implemented a 40 million MCMC chain after a burn-in of 10 million, sampling every 4000 iterations. Upon validation of the models using trace plots, we assessed four hyperparameters: 1) the proportion of phenotypic variance explained by the genotypes (PVE), the proportion of variance explained by major effect loci (PGE), 3) the posterior number of SNPs that have a sparse effect (i.e. a detectable large effect) and 4) the posterior inclusion probability (i.e. the frequency a SNP is estimated to have a detectable large effect in the MCMC, a measure of the strength of association of a SNP with a phenotype).We also predicted IOI values in GEMMA using a leave-one-out cross validation approach in a

(using the *-predict 1* flag). This was achieved by excluding the phenotypic information of one individual at a time and using the remaining 86 individuals to predict its phenotype based on their genotype. We then estimated the predictive performance of the inferred IOI by fitting a linear model to assess the proportion of observed IOI variance explained by the predicted IOI values.

Supplementary Methods 7 - Estimating timing of admixture in the contact zone

Following Johnson et al., 2011[30] and vonHoldt et al., 2022[31], we counted the number of switches in ancestry blocks from each individual, and then computed the number of generations since admixture from B = (0.04) * T*L*z(1 − z). Here, B is the number of ancestry switches, T the number of generations since admixture, L represents the genome length in cM (3782.04 cM for autosomal length and 88.37 cM for the Z chromosome) and z represents the autosomal or Z proportion of pusillus ancestry. We finally converted the obtained generation times into calendar years by multiplying the estimated number of generations since the admixture event by the generation times estimated for tinkerbirds[32]. We warn, however, that given the lack of life history data for tinkerbirds, generation times for tinkerbirds may have been estimated and, as a consequence, estimated admixture times may be slightly biased towards more recent admixture times.

Supplementary Methods 8 - Hybrid song instability models and post hoc test

For the Double Hierarchical Generalized Linear Models we performed tests using 86 of the 87 individuals recorded in the contact zone. We removed one juvenile hybrid individual whose inconsistent song we deemed an age-related factor. Specifically, while this song sounded almost like a typical adult song, it had an alternating rhythm which in recent field expeditions we have found to be emitted by young birds, and given that this individuals was also admixed (AS28707, proportion *pusillus* ancestry ~0.30) we decided upon its removal since its inclusion could have biased our results. Model selection was then based on an information criterion approach. We compared the widely applicable information criterion (WAIC) for three models. The first model included only the linear effect of ancestry in the variance part to account for the possibility that one species may be more variable than another, as recently found in zebra finches[68]. The second model was fitted with just the quadratic effect of ancestry to test for non linear relationships

between ancestry and IOI (i.e. instability of hybrid individuals), and a final full model included both terms.

Quadratic effects can occur due to effects occurring prior to the inflection point of the quadratic curve, effects after the inflection point of the quadratic curve, or both (i.e. here an increase or decrease in IOI variance with higher proportions of *extoni* ancestry, *pusillus* ancestry, or both). We therefore estimated the value of IOI variance at the statistical peak (IOI RWV peak, the *y* coordinate of the inflection point of the quadratic function) and its corresponding ancestry value (ancestry at peak, the *x* coordinate of the inflection point of the quadratic effect)[33] to then perform a pre-/post-peak analysis. With β_0 being the intercept of the RWV part of the model (also called σ), β_1 the variance (σ) estimate of the linear effect and β_2 the σ estimate of the quadratic effect, we calculated the IOI RWV peak as $\beta_0 - \beta_1^2/4\beta_2$ as well as the ancestry value at the peak ($-\beta_1/2\beta_2$)[34]. We then replaced the quadratic term in the original model with two new covariates: 1) a categorical 'pre-peak' variable (with post-peak ancestry values coded as "0" and pre-peak values coded as "1") and 2) the interaction between 'pre-peak' and linear ancestry. The effect of ancestry in this *post hoc* test represents the post-peak ancestry effect, whereas the estimates of the interaction term represent the pre-peak effect as a deviation from the post-peak ancestry effect[33,35]. The sum of the two represents the pre-peak ancestry effect. After scaling all variables to aid model convergence, we ran all the above models on five chains, each with 5500 iterations (500 warm-up) and maximal tree depth set to 15.

Supplementary Methods 9 - Quantification of playback effects

The Southern African yellow- (*P. c. extoni*) and red-fronted (*P. p. pusillus*) tinkerbird are elusive birds that tend to inhabit the canopy cover of woodland savannas and scarp forest, respectively. It is therefore extremely difficult to attract them to the mist nets unless conspecific playbacks are used. This approach is especially advantageous at the beginning of the breeding season, when the territorial response of the breeding pairs is at its peak. However, we acknowledge that using playbacks may bias some acoustic parameters. For this reason, we attempted to quantify the effect that playbacks have on tinkerbird vocalizations by comparing measurements obtained from recordings pre- and post-playback. To investigate potential effects of playback, we recorded seven *extoni* individuals in allopatry before and after playing their conspecific song and compared their measurements using a two-tailed t-test. Even though we acknowledge that our

sample size is limited, we observed a high degree of overlap between pre- and post-playback acoustic measurements, which are statistically non-significant ($t = -0.55901$, $df = 8.825$, p-value $= 0.5901$, Supplementary Fig. 12).

Supplementary Methods 10 - Estimation of repeatability

One of the most important assumptions underlying this study is that IOI is repeatable (i.e. consistent within individuals). We therefore quantified repeatability (intra-class correlation coefficient) in IOI using the *rptR* package in R v 0.9.22[36]. After performing 1000 parametric bootstraps, we estimated a repeatability score of 0.91 (95% CI: 0.89 - 0.93). Therefore, a substantial proportion of the variation in IOI (~90%) can be attributed to consistent differences among individuals, and that IOI is consistent across multiple IOI measurements of each individual. This result suggests that IOI is likely to be influenced by inherent characteristics of the recorded individuals, rather than by external factors, thus reflecting the innate nature of IOI in tinkerbirds.

Supplementary Methods 11 - Assessing covariance of phenotypes

A preference for faster and more stable songs could be responsible for the observed patterns, with pure *pusillus* females tending to mate with males with a higher proportion of *pusillus* ancestry, which sing faster and more stable songs. However, the observed pattern of asymmetric hybridization may arise as a consequence of multiple factors, which can act individually or in synergy. Therefore, to better understand the potential causes of this mating pattern, we estimated Pearson's correlation between body size, song frequency and forecrown plumage color with IOI, since covariation of two or more of these traits may conceal the relative importance of a trait over the other. We show that IOI is not strongly correlated with body size ($r = 0.17$) and song frequency ($r = 0.28$). On the other hand, we reveal a stronger correlation with Hue ($r = 0.65$), a measure of plumage coloration. This is not surprising since *extoni*, which sings on average slower songs, also has yellow forecrown, and *pusillus*, with red forecrown, has a faster IOI.

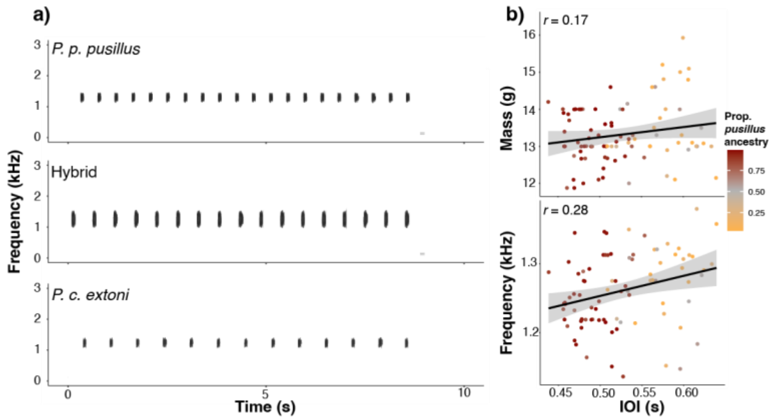

Figure S1. Spectrograms of tinkerbird song and relationships between IOI, frequency and mass. Figure illustrating **a)** full spectrograms of the 3 recordings shown in Figure 1b as well as **b)** correlations between IOI and body mass (upper panel) and IOI and peak frequency (lower panel), with *r* values denoting Pearson's correlation. Conditional mean and 95% CI are shown in panels **b)** and **c)**.

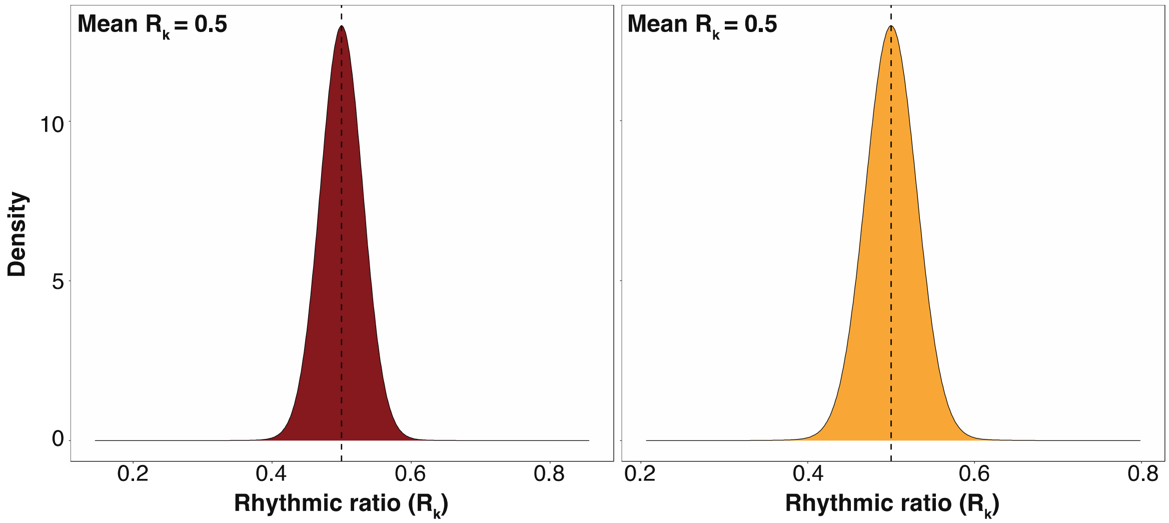

Figure S2. Tinkerbird song is isochronous. Rhythmic ratios of *P. pusillus* (left, n = 43) and *extoni* (right, n = 81) showing that the IOI intervals of their songs generate the 1:1 on integer ratio, therefore showing isochrony in tinkerbirds.

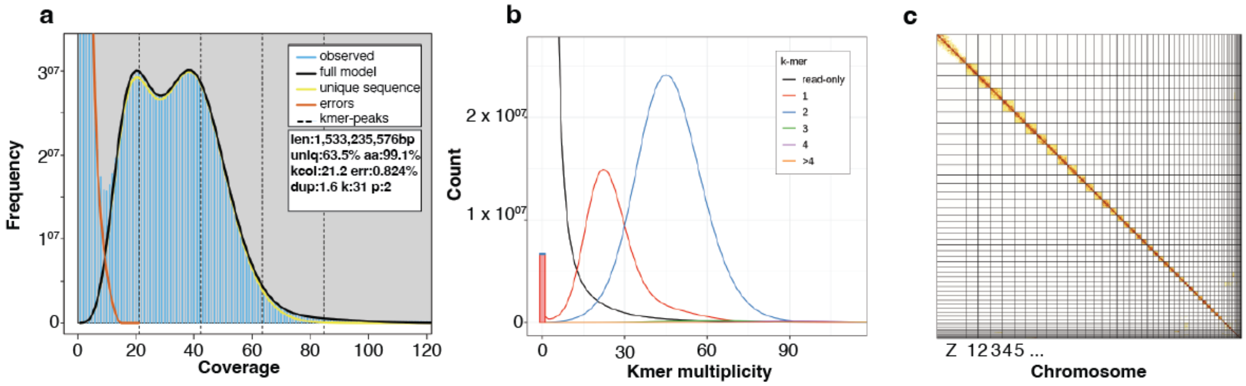

Figure S3. Genome assembly quality control. (**a**) Genomescope 2.0 [37] *k*-mer profile generated from unassembled 10x linked-reads data of the reference genome individual. The x axis reports multiplicity of *k*-mers in the raw reads and the y axis reports their cumulative frequency. The figure also reports the estimated genome size, repeat content, duplication content and heterozygosity. (**b**) Merqury[38] spectra-cn plot of the reference genome using the primary and alternate assembly, and 10x linked-reads. Similarly to panel (**a**), the x axis reports *k*-mer multiplicity and the y-axes their frequency in the reads. The curves discriminate the *k*-mers occurrence in the assemblies, while the black bar at the origin of the graph shows *k*-mers found only in the assemblies (putative assembly errors). Two peaks are visible: the red one is the haploid peak (k-mers found only one in the assemblies) and the blue is the diploid peak (k-mer found twice in the assemblies), occuring at half average coverage and at the average coverage, respectively. No duplicated *k*-mers are reported (green, purple and yellow curves). (**c**) Hi-C interaction heatmap for the chromosome-level reference genome. Both axes represent the assembly, while the diagonal reports the 3D proximity of interacting Hi-C read pairs among the assembly, with their strength being represented by color intensity. A scaffold is considered a well-assembled chromosome when the diagonal is strong and there are no off-diagonal interactions. Chromosomes are ordered in decreasing length and the name of the biggest chromosomes are shown at the bottom of the heatmap.

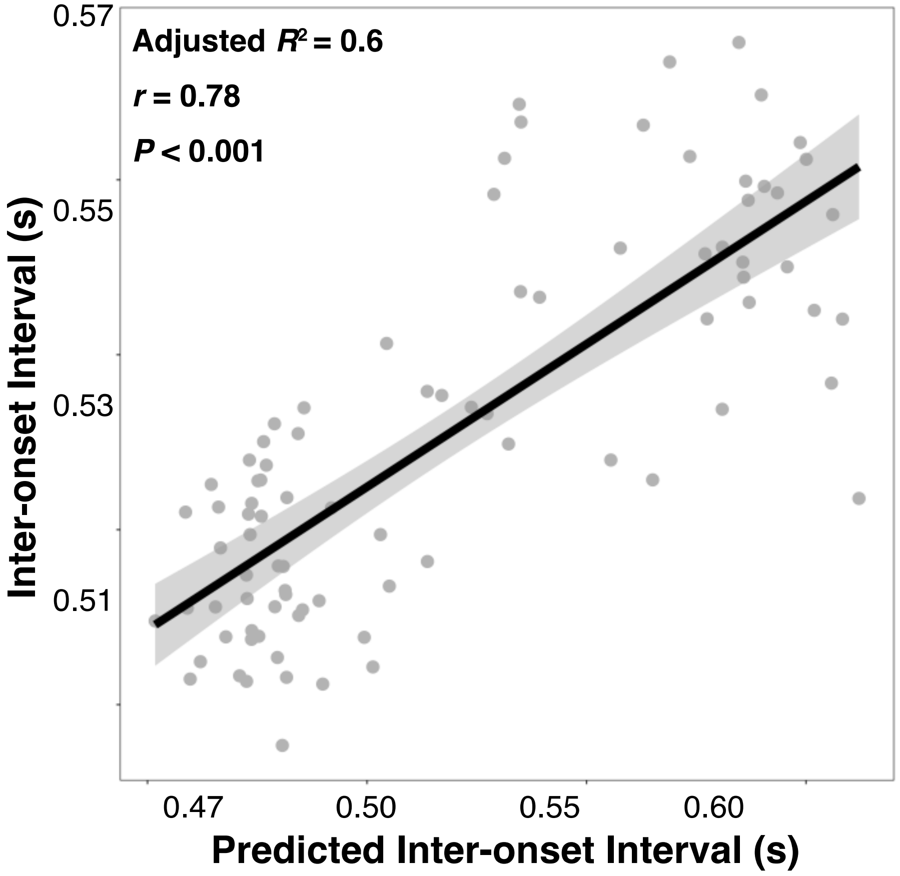

Figure S4. IOI can be reliably predicted by the genomic dataset. Observed phenotype versus phenotype predicted with Bayesian sparse linear mixed model in GEMMA for 86 sympatric individuals. The proportion of variance explained by the predicted phenotype (adjusted R^2), Pearson correlation (r) and significance value from a linear model (P) are also shown. Gray shades indicate 95% CI for the predicted mean values from the linear model.

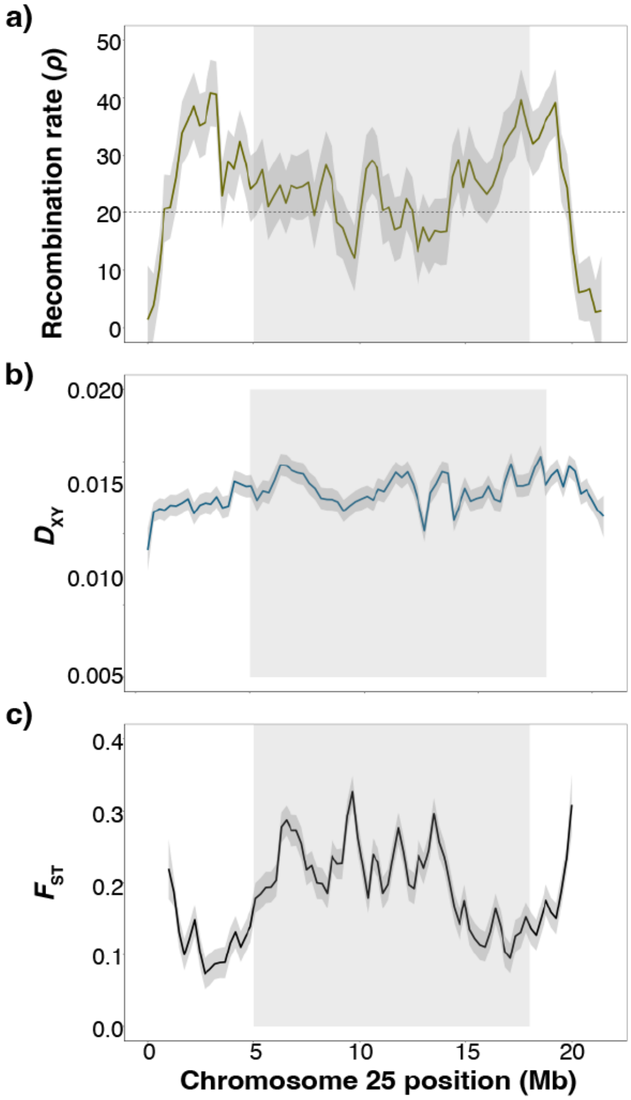

Figure S5. Diversity scans and recombination rates. Genomic scans for chromosome 25 illustration **a)** recombination rates estimated based on 26 allopatric *extoni* as well as genomic scans of **b)** absolute and **c)** relative differentiation between > 95% pure ancestry *extoni* and *pusillus* in sympatry. The gray shaded area spans the range of the significant SNPs.

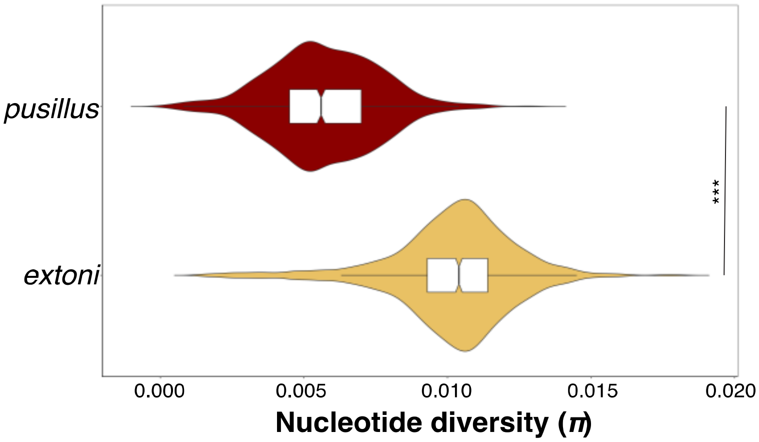

Figure S6. Genetic variability in tinkerbirds. Comparison of nucleotide diversity (**π**) across chromosome 25 between *extoni* (n = 28) and *pusillus* (n = 11). *** P < 0.001 (see Table S9).

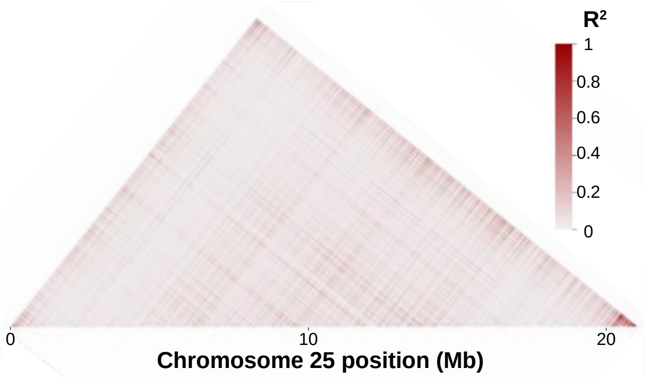

Figure S7. Chromosome 25 is characterised by low linkage. Linkage disequilibrium across chromosome 25 of the sympatric individuals

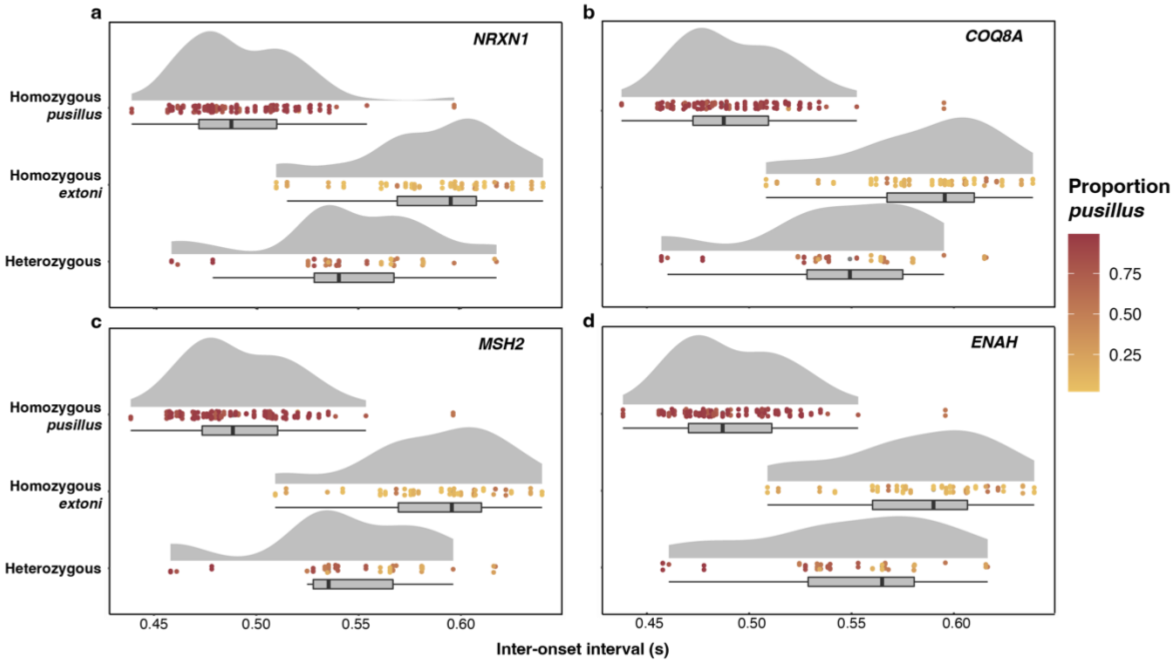

Figure S8. Additive allelic effects on IOI. Genotypes of the significant SNPs identified in GEMMA for A) *NRXN1*, B) *COQ8A*, C) *MSH2* and D) *ENAH* plotted against the inter-onset interval (IOI) of both allopatric and sympatric individuals (n = 138). Dot colors reflect genome-wide ancestry. The central line within each boxplot represents the median, with box limits representing the 1st (Q1) and 3rd quartile (Q3). Whiskers are calculated as Q1/Q3 -/+ 1.5 * (Interquartile range).

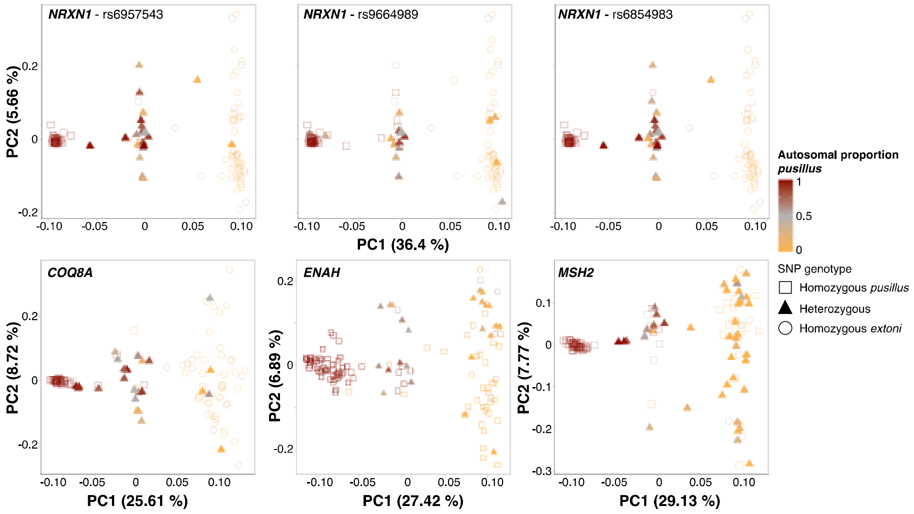

Figure S9. Gene-specific SNPs cluster in three groups. PC1 to PC2 plots of the GEMMA significant SNPs that mapped into specific genes on chromosome 25.

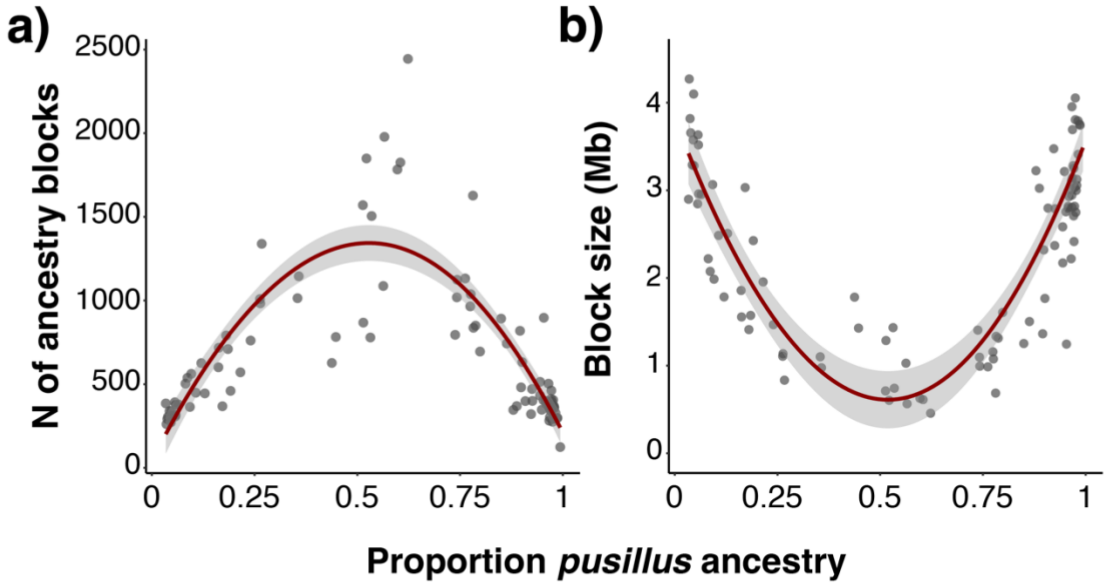

Figure S10. Ancestry blocks and tinkerbird ancestry. Plot showing variation of **a)** the number of ancestry blocks and **b)** block size with proportion of *pusillus* ancestry in sympatric individuals. Red smooth lines represent the conditional means, whereas 95% CI are represented in gray.

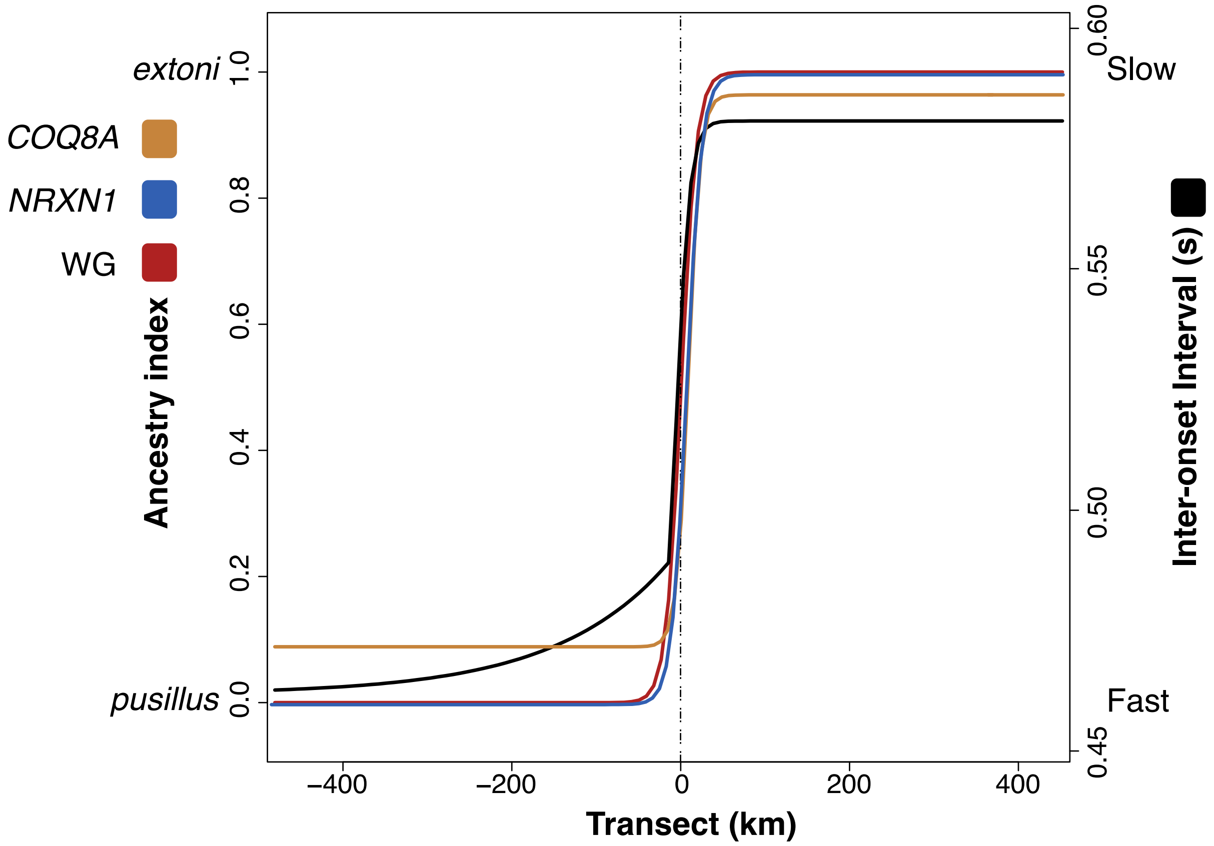

Figure S11.Geographic clines of ancestry and IOI Geographic clines illustrate concordant trends in ancestry across the entire genome (red), within *NRXN1* (blue), *COQ8A* (ochre) as well as IOI (black) across the contact zone.

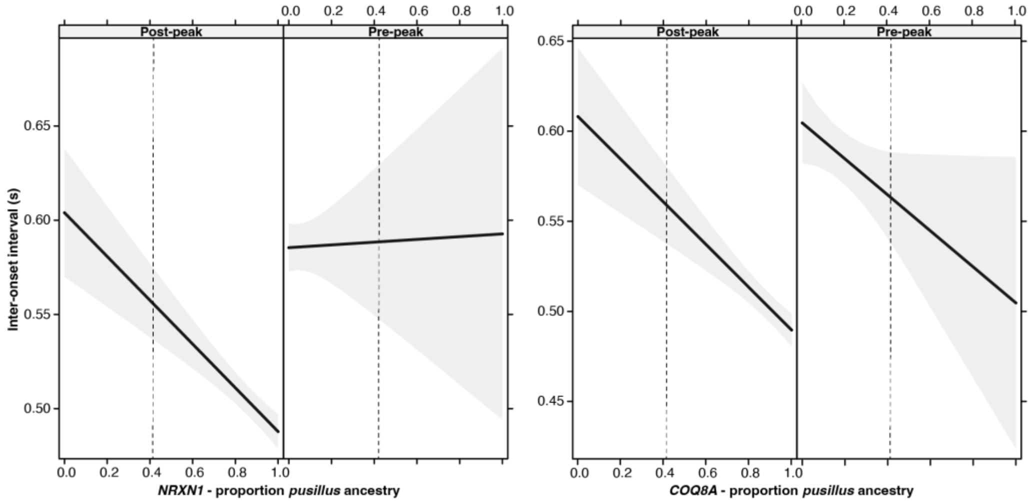

Figure S12. IOI stability *post-hoc* analysis. Model effects of the *post hoc* test to investigate effects of ancestry within (**A**) *NRXN1* and (**B**) *COQ8A* on the residual within-individual variance in inter-onset interval for 86 sympatric individuals. The dashed line demarks the ancestry value at the performance peak. For each panel, only the respective pre- (< than the ancestry value at the peak; left of dashed line) or post-peak effects (> than the ancestry value at the peak; right of dashed line) are relevant for interpretation. Mean IOI and its variance (95% CI) decrease with increasing *pusillus* ancestry in both genes after the peak. Pre-peak the effects differ between the two genes, with again a significant decrease in IOI with increasing pusillus ancestry at COQ8A, but stable mean IOI, yet with increasing variance, with increasing pusillus ancestry at NRXN1.

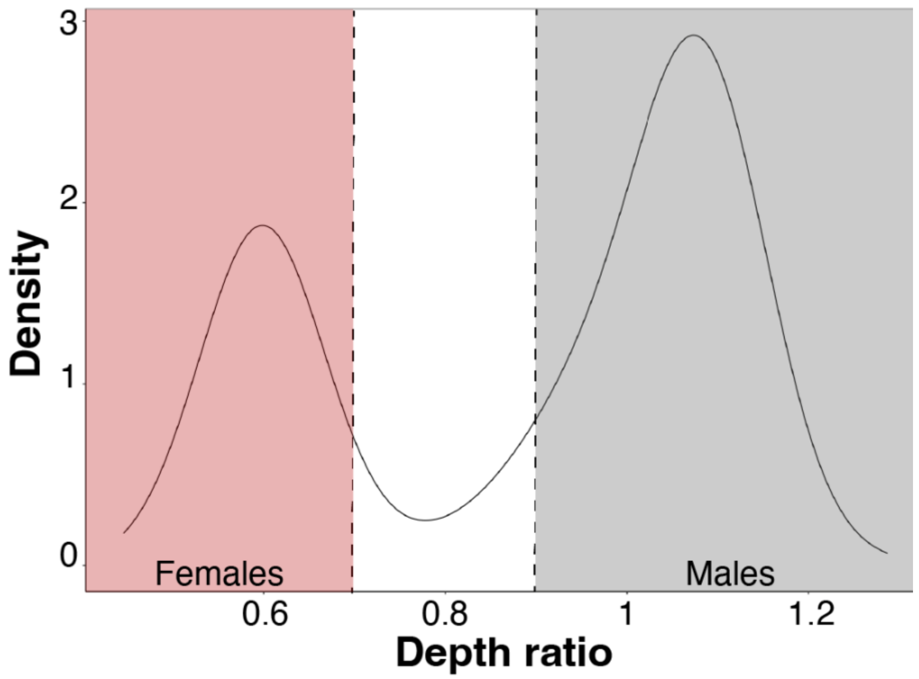

Figure S13. Tinkerbird sexing by sequencing depth. Distribution of the depth ratio of 452 tinkerbirds sequenced with ddRAD.

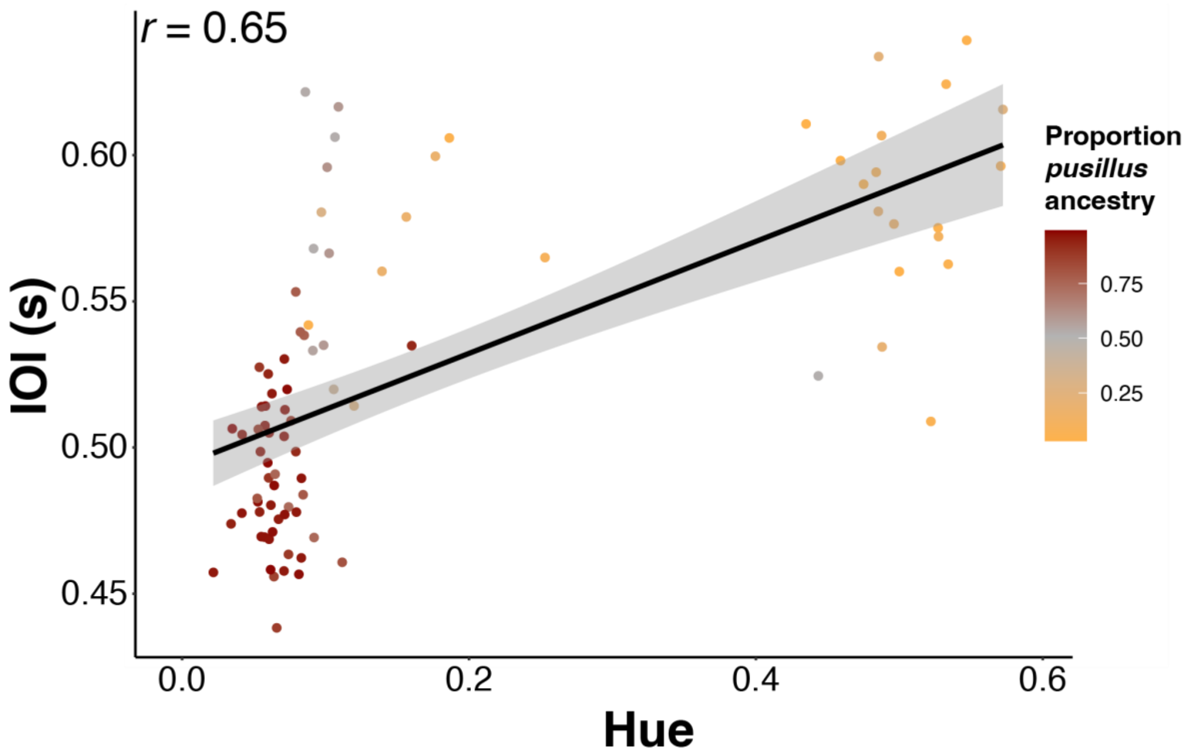

Figure S14. Relationship between IOI and forecrown feather hue in 87 tinkerbirds from the contact zone. Hue values < 0.2 indicate red forecrown coloration, Hue > 0.2 and < 0.4 reflects orange - amber forecrowns, whereas high values of Hue represent yellow forecrowns. The plot reveals that not all individuals with red forecrowns (Hue < 0.2) have a higher proportion of *pusillus* ancestry, whereas the individuals with fast songs (IOI < 0.5) are mostly pure *pusillus*. The *r* score indicates Pearson's correlation. Mean fitted values and their 95% CI are represented by the black line and gray shaded areas, respectively.

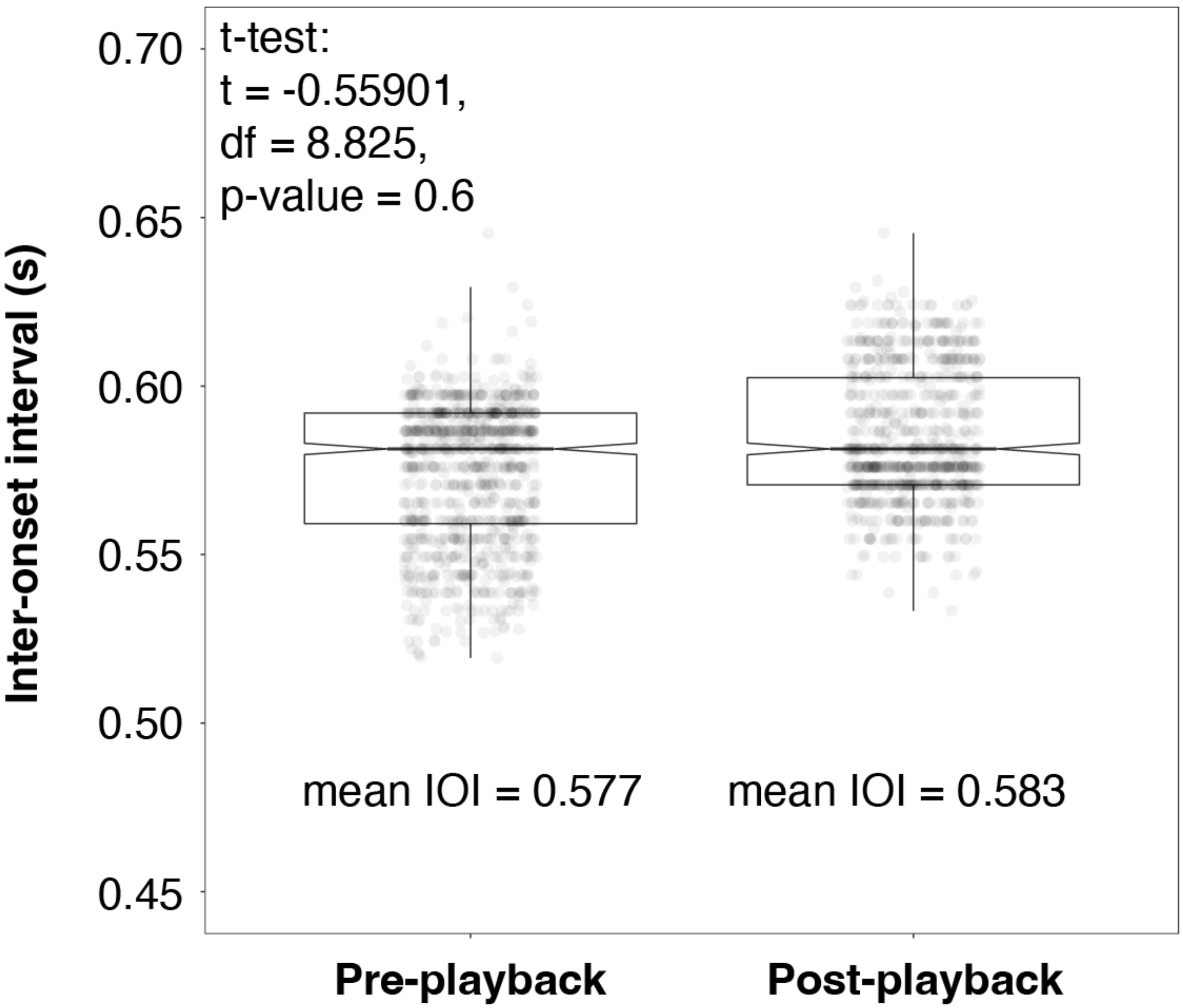

Figure S15. Effect of playback on tinkerbird IOI. Vocal response of seven individuals of *P. chrysoconus extoni* recorded in allopatry pre- and post- playback. The plot shows high overlap between IOI measurements obtained before and after exposing the singing birds to conspecific playbacks which show no statistical differences. Test statistics refer to a two-tailed t-test. The central line within each boxplot represents the median, with box limits representing the 1st (Q1) and 3rd quartile (Q3). Whiskers are calculated as Q1/Q3 -/+ 1.5 * (Interquartile range).

Table S1. Genomescope 2.0 assembly predictions (31 bp *k*-mer size) based on unassembled 10x linked reads.

**Property**	**Min**	**Max**
Homozygous (aa)	99.05%	99.06%
Heterozygous (ab)	0.94%	0.95%
Genome Haploid Length (bp)	1,530,334,502	1,533,235,576
Genome Repeat Length (bp)	558,598,976	559,657,918
Genome Unique Length (bp)	971,735,527	973,577,657
Model Fit	65.99%	99.34%
Read Error Rate	0.82%	0.82%

Table S2. Assembly statistics measured with gfastats

**Metrics**	**Scaffolds**	**Contigs**	**Gaps**
Total length (bp)	1,272,338,968	1,266,326,211	6,012,757
Average length (bp)	5,048,964.16	2,265,342.06	19,585.53
Largest (bp)	117,909,544	47,589,575	824,725
NG50	26,323,978	12,736,711	-
N50	33,994,490	16,792,259	173,817
N90	14,595,720	2,442,898	67,291

Table S3. QV and *k*-mer completeness measured with Merqury using a 21 *k*-mer length Meryl database.

**Merqury metrics**	**Primary assembly**	**Alternate assembly**	**Both**
QV	428.711	363.615	386.947
k-mer completeness	85.2	756.535	939.333

Table S4. Functional completeness measured with BUSCO using the Vertebrata orthologous genes database. Busco search results: C:95.2% [S:94.6%, D:0.6%], F:1.1%, M:3.7%, n:3354.

BUSCO category	Number of genes found in the assembly	% of genes found in the assembly
Complete BUSCOs (C)	3191	95.2
Complete and single-copy BUSCOs (S)	3172	94.6
Complete and duplicated BUSCOs (D)	19	0.6
Fragmented BUSCOs (F)	37	1.1
Missing BUSCOs (M)	126	3.7
Total BUSCO groups searched (n)	3354	100

Table S5. Complete list of the significant SNPs associated with Inter-Onset Interval (IOI) highlighted by the association test in GEMMA.

SNP ID	tinkerbird (chr25)	equivalent in zebra finch (chr3)	plog	Gene	Function
rs1	rs5022427	rs31906116	6.338	Maps 24089 bp downstream (3') of LOC121469595	
rs2	rs6851380	no alignment	6.024	-	
rs3	rs6854983	rs91286018	6.089	*NRXN1*	Plasticity of synaptic adhesion and signaling between neurons [39,40]. Associated with speech disorders in humans [41–43] and migration in passerines [44,45].
rs4	rs6957543	rs91357874	7.031	*NRXN1*	Plasticity of synaptic adhesion and signaling between neurons [39,40]. Associated with speech disorders in humans [41–43] and migration in passerines [44,45].
rs5	rs7737388	rs94412127	6.229	*COQ8A*	Biosynthesis of COQ10, responsible for generating cellular ATP [46]. Mutations in this gene are associated with vocal impairments in humans [47–49].
rs6	rs8886686	rs92848648	6.071	Maps 817 bp downstream (3') of *ALK*	Cellular growth and division [50].
rs7	rs9449012	rs92405353	7.404	*MSH2*	Responsible for correcting DNA replication errors[1]. Deficiencies of MSH2 lead to increased cancer risk [51,52].
rs8	rs9589214	rs92269412	6.545	Maps 48084 bp upstream (5') of *MSH6*	
rs9	rs9602675	rs92263920	6.983	Maps 56857 bp upstream (5') of *MSH6*	
rs10	rs9664989	rs91133082	6.612	*NRXN1*	Plasticity of synaptic adhesion and signaling between neurons [39,40]. Associated with speech disorders in humans [41–43] and migration in passerines [44,45].
rs11	rs9791236	rs92074731	6.587	Maps 15296 bp upstream (5') of *FOXN2*	Proliferation and neuronal growth [53].

rs12	rs9797804	rs92068897	6.588	Maps 9462 bp upstream (5’) of *FOXN2*	Proliferation and neuronal growth [53].
rs13	rs10249778	rs98821956	6.354	Maps 98291 bp upstream (5’) of *EML4*	Organization and stabilization of cellular microtubules. Is a fusion partner of *ALK* to form an oncogene responsible for lung cancer [54,55].
rs14	rs11568784	rs100142032	6.085	Maps 69 bp upstream (5’) of LOC115494518	
rs15	rs17821223	rs95030683	6.056	*ENAH*	Neuronal development and formation of dendritic spines [56]. Mutations of this gene can affect cognitive processes [57].

[1]Human Protein Atlas: https://www.proteinatlas.org/

Table S6. Genomic features of the areas where the significant SNPs are located obtained from the zebra finch reference genome (bTaeGut1.4.pri) general feature format (GFF) file. For *COQ8A*, the start and end of the exon are shown within which the significant SNP is found.

**Gene**	**SNP**	**Region**	**Exon preceding**	**Exon succeeding**
NRXN1	rs91286018	intron 15	91274007 - 91274188	91362532 - 91362703
NRXN1	rs91357874	intron 15	91274007 - 91274188	91362532 - 91362703
NRXN1	rs91133082	intron 12	91127988 - 91128178	91143267 - 91143440
MSH2	rs92405353	intron 6	92397479 - 92397678	92415614 - 92415747
ENAH	rs95030683	intron 1	94986766 - 94987081	95030789 - 95030954
COQ8A	rs94412127	exon 15	94411877	94413237

Table S7. Two-sided t-test output table comparing the standard deviation over the mean, a measure of variance explained, between 10000 randomly selected SNPs across the genome and the four genes highlighted by the study.

**Region 1**	**Region 2**	***t***	***df***	***p***
WG 10K	NRXN1	-41.54	25034	<0.001
WG 10K	COQ8A	-17.03	1116	<0.001
WG 10K	MSH2	-18.90	1460	<0.001
WG 10K	ENAH	-2.87	1432	0.004

Table S8. GLMM output showing the effect of vegetation density, represented by the Enhanced Vegetation Index, and autosomal proportion of *pusillus* ancestry on IOI for 87 sympatric individuals. Since no contrasts are applied, no adjustments for multiple comparisons are applied.

**Response: IOI**	**β**	**Std. Error**	***z***	***p***
**a)**				
Intercept	0.057	0.065	0.872	0.383
EVI	-0.102	0.066	-1.548	0.122
Autosomal prop. *P. pusillus*	**-0.774**	**0.066**	**-11.690**	**<0.001**

Table S9. Two-sided t-test results comparing mean (± sd) nucleotide diversity (π) between *extoni* and *pusillus* allopatric populations.

**Statistic**	**Population**	***P. chrysoconus***	***P. pusillus***	***t***	***df***	***p***
π	allopatric	0.010 (± 0.002)	0.005 (± 0.001)	44.15	1594.5	< 0.001

Table S10. GLMM output showing the effect of autosomal ancestry (**a**), Z-linked ancestry (**b**) and sex on the Inter-onset Interval. Significant terms are represented in bold. No adjustments for multiple comparisons were applied.

**Response: IOI**	**Estimate**	**Std. Error**	***z***	***p***
**a)**				
Intercept	**0.594**	**0.011**	**51.57**	**< 0.001**
Autosomal prop. *P. pusillus*	**-0.112**	**0.009**	**-11.70**	**< 0.001**
Males	0.005	0.011	0.46	0.644
**b)**				
Intercept	**0.595**	**0.012**	**47.58**	**< 0.001**
Z-linked prop. *P. pusillus*	**-0.101**	**0.009**	**-10.28**	**< 0.001**
Males	-0.001	0.012	-0.12	0.902

Table S11. DHGLM output on IOI instability of hybrid individuals (n = 86) at the contact zone (H1). Three models testing the effect of whole-genome ancestry (**a** – **c**) are compared using WAIC scores, with the best model being the one with the lowest WAIC value. We then tested for the effects on IOI of varying ancestry in specific loci: **d**) *NRXN1*, **e**) *COQ8A*, **f**) *MSH2* and **g**) *ENAH*. Significant terms are represented in bold.

**Response: IOI**	**Estimate**	**Std. Error**	**LCL**	**UCL**	**Rhat**
**a)**					
**WAIC:** 20724.63					
Intercept	**0.15**	**0.07**	**0.01**	**0.28**	**1.02**
σ Intercept	**-1.31**	**0.04**	**-1.39**	**-1.22**	**1.01**
Prop. *P. pusillus*	**-0.84**	**0.07**	**-0.98**	**-0.70**	**1.01**
σ prop. *P. pusillus*	**-0.10**	**0.04**	**-0.19**	**-0.02**	**1.01**
**b)**					
**WAIC:** 20756.01					
Intercept	**0.15**	**0.07**	**0.01**	**0.28**	**1.00**
σ Intercept	**-1.29**	**0.09**	**-1.47**	**-1.12**	**1.01**
Prop. *P. pusillus*	**-0.83**	**0.07**	**-0.97**	**-0.69**	**1.01**
σ prop. *P. pusillus*^{2}	-0.04	0.09	-0.21	0.14	1.01
**c)**					
**WAIC:** 20756.96					
Intercept	**0.15**	**0.07**	**0.02**	**0.28**	**1.01**
σ Intercept	-1.22	0.09	1.41	-1.04	1.00
Prop. *P. pusillus*	**-0.83**	**0.07**	**-0.98**	**-0.69**	**1.00**
σ prop. *P. pusillus*^{2}	-0.09	0.09	-0.27	0.08	1.00

σ prop. *P. pusillus*	**-0.12**	**0.05**	**-0.21**	**-0.02**	**1.00**
**d)**					
Intercept	-0.06	0.07	-0.20	0.06	1.01
σ Intercept	**-1.11**	**0.11**	**-1.33**	**-0.89**	**1.00**
NRXN1 prop. *P. pusillus*	-0.73	0.06	-0.86	-0.60	1.00
σ NRXN1 prop. *P. pusillus*^{2}	**-0.21**	**0.10**	**-0.40**	**-0.02**	**1.00**
σ NRXN1 prop. *P. pusillus*	**-0.21**	**0.09**	**-0.38**	**-0.04**	**1.00**
**e)**					
Intercept	-0.06	0.07	-0.19	0.07	1.01
σ Intercept	**-1.12**	**0.11**	**-1.34**	**-0.89**	**1.00**
COQ8A prop. *P. pusillus*	**-0.73**	**0.06**	**-0.86**	**-0.60**	**1.01**
σ COQ8A prop. *P. pusillus*^{2}	**-0.20**	**0.10**	**-0.40**	**-0.01**	**1.00**
σ COQ8A prop. *P. pusillus*	**-0.21**	**0.09**	**-0.38**	**-0.04**	**1.00**
**f)**					
Intercept	-0.06	0.06	-0.18	0.07	1.00
σ Intercept	**-1.12**	**0.12**	**-1.36**	**-0.90**	**1.00**
MSH2 prop. *P. pusillus*	**-0.75**	**0.06**	**-0.87**	**-0.63**	**1.00**
σ MSH2 prop. *P. pusillus*^{2}	-0.20	0.11	-0.41	0.01	1.00
σ MSH2 prop. *P. pusillus*	**-0.20**	**0.09**	**-0.36**	**-0.03**	**1.00**

g)

Intercept	-0.05	0.07	-0.18	0.09	1.01
σ Intercept	**-1.25**	**0.09**	**-1.42**	**-1.08**	**1.00**
ENAH prop. *P. pusillus*	**-0.72**	**0.07**	**-0.85**	**-0.59**	**1.01**
σ ENAH prop. *P. pusillus*^{2}	-0.08	0.07	-0.21	0.06	1.00
σ ENAH prop. *P. pusillus*	**-0.13**	**0.06**	**-0.24**	**-0.02**	**1.01**

Table S12. Table illustrating the post-hoc analysis of DHGLM models with a significant quadratic effect based on *NRXN1* and *COQ8A* ancestry, with panel (a) showing peak values (and their estimates (β) back-transformed to the original scale for IOI) and (b) the results of the pre- and post-peak analysis.

	**NRXN1**	**COQ8A**
**a)**	**β (95% CI)**	**β (95% CI)**
IOI at peak (95% CI)	-1.05 (-1.29/ -0.81)[1]	-1.05 (-1.32/ -0.81)[2]
Ancestry at peak (95% CI)	-0.49 (-1.13/- 0.24)[3]	-0.49 (-1.13/- 0.24)[3]
**b)**		
Pre-peak ancestry effect (95% CI)	0.06 (-0.69; 0.83)	-0.65(-1.30; -0.03)
Post-peak ancestry effect (95% CI)	-0.87 (-1.16; -0.59)	-0.75 (-1.01; -0.49)

[1] Backscaled β (95% CI) = 0.47 (0.45 – 0.48)

[2] Backscaled β (95% CI) = 0.47 (0.45 – 0.48)

[3] Backscaled β (95% CI) = 0.43 (0.18 – 0.54)

[4] Backscaled β (95% CI) = 0.43 (0.18 – 0.54)

Table S13. DHGLM output testing for character displacement (H2) in IOI as well as its variance in pure parental-species (> 99% ancestry of *extoni* or *pusillus*) individuals (n = 181). Significant terms are in bold.

**Response: IOI**	**Estimate**	**Std. Error**	**LCL**	**UCL**	**Rhat**
Intercept	**0.38**	**0.05**	**0.28**	**0.48**	**1.03**
σ Intercept	**-1.61**	**0.06**	**-1.72**	**-1.50**	**1.01**
P. pusillus	**-1.61**	**0.08**	**-1.78**	**-1.44**	**1.03**
Sympatry	**0.15**	**0.02**	**0.11**	**0.18**	**1.00**
P. pusillus x sympatry	**0.47**	**0.10**	**0.28**	**0.67**	**1.02**
σ *P. pusillus*	-0.15	0.09	-0.34	0.04	1.01
σ sympatry	**0.98**	**0.05**	**0.89**	**1.08**	**1.00**
σ *P. pusillus* x sympatry	**-0.67**	**0.11**	**-0.89**	**-0.45**	**1.00**

Table S14. Output of a linear model (LM) showing a significant difference in the level of assortative mating between putatively pure *extoni* mothers and putatively pure *pusillus* mothers of females in the contact zone. The difference in the respective ancestry (**Δ parental ancestry**) of those mothers and the fathers that sired the females we sampled is greater in *extoni* than in *pusillus*, suggesting *pusillus* females mate assortatively with *pusillus* males but *extoni* females do not mate assortatively. No adjustments for multiple comparisons were applied.

**Response: Δ parental ancestry**	**Estimate**	**Std. Error**	***z***	***p***
Intercept	0.045	0.059	0.767	0.449
Pure *extoni* (97%)	0.582	0.086	6.714	<0.001

Table S15. DHGLM output showing no differences in IOI, its residual within-individual variance (RWV, i.e. σ) between sexes in tinkerbirds.

**Response: IOI**	**Estimate**	**Std. Error**	**LCL**	**UCL**	**Rhat**
Intercept	0.34	0.35	-0.35	1.03	1.00
**σ Intercept**	**-1.33**	**0.14**	**-1.61**	**-1.06**	**1.00**
Sex: males	-0.27	0.37	-0.99	0.45	1.00
σ sex: males	-0.02	0.15	-0.30	0.26	1.00

References

1. Kirschel, A. N. G. *et al.* CYP2J19 mediates carotenoid colour introgression across a natural avian hybrid zone. *Mol. Ecol.* **29**, 4970–4984 (2020).
2. Maia, R., Eliason, C. M., Bitton, P.-P., Doucet, S. M. & Shawkey, M. D. pavo: an R package for the analysis, visualization and organization of spectral data. *Methods Ecol. Evol.* **4**, 906–913 (2013).
3. Chin, C.-S. *et al.* Nonhybrid, finished microbial genome assemblies from long-read SMRT sequencing data. *Nat. Methods* **10**, 563–569 (2013).
4. Chin, C.-S. *et al.* Phased diploid genome assembly with single-molecule real-time sequencing. *Nat. Methods* **13**, 1050–1054 (2016).
5. Guan, D. *et al.* Identifying and removing haplotypic duplication in primary genome assemblies. *Bioinformatics* **36**, 2896–2898 (2020).
6. Formenti, G. *et al.* SMRT long reads and Direct Label and Stain optical maps allow the generation of a high-quality genome assembly for the European barn swallow (Hirundo rustica rustica). *Gigascience* **8**, (2019).
7. Ghurye, J. *et al.* Integrating Hi-C links with assembly graphs for chromosome-scale assembly. *PLoS Comput. Biol.* **15**, e1007273 (2019).
8. Rhie, A. *et al.* Towards complete and error-free genome assemblies of all vertebrate species. *Nature* **592**, 737–746 (2021).
9. Formenti, G. *et al.* Complete vertebrate mitogenomes reveal widespread gene duplications and repeats. *Genome Biol* **22**, (2021).
10. Garrison, E. & Marth, G. Haplotype-based variant detection from short-read sequencing. *arXiv [q-bio.GN]* (2012).
11. Howe, K. *et al.* Significantly improving the quality of genome assemblies through curation. *Gigascience* **10**, (2021).

12. Chow, W. *et al.* gEVAL — a web-based browser for evaluating genome assemblies. *Bioinformatics* **32**, 2508–2510 (2016).

13. Harry, E. *PretextView (Paired REad TEXTure Viewer): A desktop application for viewing pretext contact maps. 2022;(Accessed: 19 October 2022)*. (2022).

14. Formenti, G. *et al.* Gfastats: conversion, evaluation and manipulation of genome sequences using assembly graphs. *bioRxiv* 2022.03.24.485682 (2022) doi:10.1101/2022.03.24.485682.

15. Rhie, A., Walenz, B. P., Koren, S. & Phillippy, A. M. Merqury: reference-free quality, completeness, and phasing assessment for genome assemblies. *Genome Biol.* **21**, 245 (2020).

16. Simão, F. A., Waterhouse, R. M., Ioannidis, P., Kriventseva, E. V. & Zdobnov, E. M. BUSCO: assessing genome assembly and annotation completeness with single-copy orthologs. *Bioinformatics* **31**, 3210–3212 (2015).

17. Community, T. G. *et al.* The Galaxy platform for accessible, reproducible and collaborative biomedical analyses: 2022 update. *Nucleic Acids Research* Preprint at https://doi.org/10.1093/nar/gkac247 (2022).

18. Secomandi, S. *et al.* A chromosome-level reference genome and pangenome for barn swallow population genomics. *Cell Rep.* **42**, 111992 (2023).

19. Morgulis, A., Gertz, E. M., Schäffer, A. A. & Agarwala, R. WindowMasker: window-based masker for sequenced genomes. *Bioinformatics* **22**, 134–141 (2006).

20. Tarailo-Graovac, M. & Chen, N. Using RepeatMasker to identify repetitive elements in genomic sequences. *Curr. Protoc. Bioinformatics* **Chapter 4**, Unit 4.10 (2009).

21. Bao, W., Kojima, K. K. & Kohany, O. Repbase Update, a database of repetitive elements in eukaryotic genomes. *Mob. DNA* **6**, 11 (2015).

22. Martin, M. Cutadapt removes adapter sequences from high-throughput sequencing reads. *EMBnet.journal* **17**, 10–12 (2011).

23. Houtgast, E. J., Sima, V.-M., Bertels, K. & Al-Ars, Z. Hardware acceleration of BWA-MEM genomic short read mapping for longer read lengths. *Comput. Biol. Chem.* **75**, 54–64 (2018).

24. Li, H. *et al.* The Sequence Alignment/Map format and SAMtools. *Bioinformatics* **25**, 2078–2079 (2009).

25. Broad Institute. “Picard Toolkit”. GitHub Repository. https://broadinstitute.github.io/picard. (2018)

26. McKenna, A. *et al.* The Genome Analysis Toolkit: a MapReduce framework for analyzing next-generation DNA sequencing data. *Genome Res.* **20**, 1297–1303 (2010).

27. Danecek, P. *et al.* The variant call format and VCFtools. *Bioinformatics* **27**, 2156–2158 (2011).

28. Catchen, J., Hohenlohe, P. A. & Bassham, S. Stacks: an analysis tool set for population genomics. *Molecular* (2013).

29. Altschul, S. F., Gish, W., Miller, W., Myers, E. W. & Lipman, D. J. Basic local alignment search tool. *J. Mol. Biol.* **215**, 403–410 (1990).

30. Johnson, N. A. *et al.* Ancestral components of admixed genomes in a Mexican cohort. *PLoS Genet.* **7**, e1002410 (2011).

31. vonHoldt, B. M. *et al.* Reviving ghost alleles: Genetically admixed coyotes along the American Gulf Coast are critical for saving the endangered red wolf. *Sci Adv* **8**, eabn7731 (2022).

32. Bird, J. P. *et al.* Generation lengths of the world’s birds and their implications for extinction risk. *Conserv. Biol.* **34**, 1252–1261 (2020).

33. Bouwhuis, S., Sheldon, B. C., Verhulst, S. & Charmantier, A. Great tits growing old: selective disappearance and the partitioning of senescence to stages within the breeding cycle. *Proc. Biol. Sci.* **276**, 2769–2777 (2009).

34. Bronshtein, I. N., Semendyayev, K. A., Musiol, G. & Mühlig, H. *Handbook of Mathematics*. (Springer, 2015).

35. Dingemanse, N. J., Moiron, M., Araya-Ajoy, Y. G., Mouchet, A. & Abbey-Lee, R. N. Individual variation in age-dependent reproduction: Fast explorers live fast but senesce young? *J. Anim. Ecol.* **89**, 601–613 (2020).

36. Stoffel, M. A., Nakagawa, S. & Schielzeth, H. rptR: repeatability estimation and variance decomposition by generalized linear mixed-effects models. *Methods Ecol. Evol.* **8**, 1639–1644

(2017).

37. Ranallo-Benavidez, T. R., Jaron, K. S. & Schatz, M. C. GenomeScope 2.0 and Smudgeplot for reference-free profiling of polyploid genomes. *Nat. Commun.* **11**, 1432 (2020).

38. Rhie, A., Walenz, B. P., Koren, S. & Phillippy, A. M. Merqury: reference-free quality, completeness, and phasing assessment for genome assemblies. *Genome Biol.* **21**, 245 (2020).

39. Südhof, T. C. Synaptic Neurexin Complexes: A Molecular Code for the Logic of Neural Circuits. *Cell* **171**, 745–769 (2017).

40. Cheung, A. *et al.* Neurexins in serotonergic neurons regulate neuronal survival, serotonin transmission, and complex mouse behaviors. *Elife* **12**, (2023).

41. Dean, C. & Dresbach, T. Neuroligins and neurexins: linking cell adhesion, synapse formation and cognitive function. *Trends Neurosci.* **29**, 21–29 (2006).

42. Benítez-Burraco, A., Salud Jiménez-Romero, M. & Fernández-Urquiza, M. Delving into the Genetic Causes of Language Impairment in a Case of Partial Deletion of ***NRXN1***. *Molecular Syndromology* vol. 13 496–510 Preprint at https://doi.org/10.1159/000524710 (2022).

43. Kasem, E., Kurihara, T. & Tabuchi, K. Neurexins and neuropsychiatric disorders. *Neurosci. Res.* **127**, 53–60 (2018).

44. Boss, J. *et al.* Gene expression in the brain of a migratory songbird during breeding and migration. *Mov Ecol* **4**, 4 (2016).

45. Sharma, A., Singh, D., Das, S. & Kumar, V. Hypothalamic and liver transcriptome from two crucial life-history stages in a migratory songbird. *Exp. Physiol.* **103**, 559–569 (2018).

46. Desbats, M. A., Lunardi, G., Doimo, M., Trevisson, E. & Salviati, L. Genetic bases and clinical manifestations of coenzyme Q10 (CoQ 10) deficiency. *J. Inherit. Metab. Dis.* **38**, 145–156 (2015).

47. Stefely, J. A. *et al.* Cerebellar Ataxia and Coenzyme Q Deficiency through Loss of Unorthodox Kinase Activity. *Mol. Cell* **63**, 608–620 (2016).

48. Galosi, S. *et al.* Dystonia-Ataxia with early handwriting deterioration in COQ8A mutation carriers: A case series and literature review. *Parkinsonism Relat. Disord.* **68**, 8–16 (2019).

49. Arena, I. G., Pugliese, A., Volta, S., Toscano, A. & Musumeci, O. Molecular Genetics Overview of Primary Mitochondrial Myopathies. *J. Clin. Med. Res.* **11**, (2022).

50. Mossé, Y. P. *et al.* Identification of ALK as a major familial neuroblastoma predisposition gene. *Nature* **455**, 930–935 (2008).

51. Renkonen, E. *et al.* Altered expression of MLH1, MSH2, and MSH6 in predisposition to hereditary nonpolyposis colorectal cancer. *J. Clin. Oncol.* **21**, 3629–3637 (2003).

52. Yoo, K. H., Won, K. Y., Lim, S.-J., Park, Y.-K. & Chang, S.-G. Deficiency of MSH2 expression is associated with clear cell renal cell carcinoma. *Oncol. Lett.* **8**, 2135–2139 (2014).

53. Weigel, D., Jürgens, G., Küttner, F., Seifert, E. & Jäckle, H. The homeotic gene fork head encodes a nuclear protein and is expressed in the terminal regions of the Drosophila embryo. *Cell* **57**, 645–658 (1989).

54. Mano, H. Non-solid oncogenes in solid tumors: EML4-ALK fusion genes in lung cancer. *Cancer Sci.* **99**, 2349–2355 (2008).

55. Mano, H. The EML4-ALK oncogene: targeting an essential growth driver in human cancer. *Proc. Jpn. Acad. Ser. B Phys. Biol. Sci.* **91**, 193–201 (2015).

56. Goffin, J. M. *et al.* Focal adhesion size controls tension-dependent recruitment of α-smooth muscle actin to stress fibers. *J. Cell Biol.* **172**, 259–268 (2006).

57. Shahbazi, M. *et al.* Comprehensive association analysis of speech recognition thresholds after cisplatin-based chemotherapy in survivors of adult-onset cancer. *Cancer Med.* (2022).
