# Supplementary material for: A genomic basis of vocal rhythm in birds
Source: Nat Commun. 2024 Apr 23;15:3095. doi: 10.1038/s41467-024-47305-5 (PMC11039653; doi:10.1038/s41467-024-47305-5)
Supplement: Supplementary file 3 — Description of Additional Supplementary Files [file 41467_2024_47305_MOESM3_ESM.pdf]

Description of Additional Supplementary Files

Supplementary Movie 1

Description: Pusillus_23Nov21.mp4 is a ~ 7 sec video clip of a redfronted tinkerbird (*Pogoniulus pusillus pusillus*) recorded singing on 23rd Nov 2021 by A. Kirschel using a Canon R5 camera near KwaBulawayo, KwaZulu-Natal Province, South Africa.

Supplementary Movie 2

Description: Hybrid_tinkerbird_24Nov18.mp4 is a ~ 11 sec video clip of an admixed individual (this individual was subsequently banded, sampled and recorded again), recorded singing on 24th Nov 2018 by A. Kirschel using a Canon 1D Mark IV DSLR camera, near Tshaneni, Eswatini, in the contact zone of the two species.

Supplementary Movie 3

Description: extoni_14Nov21.mp4 is a ~ 8 sec video clip of a yellowfronted tinkerbird (*Pogoniulus chrysoconus extoni*) recorded singing on 14th Nov 2021 by A. Kirschel using a Canon R5 camera in De Tweedespruit Conservancy, Gauteng Province, South Africa.

Supplementary Data 1

Description: List of whole-genome samples used in this study. The species is assigned only for allopatric individuals, whereas we provide the proportion of *pusillus* ancestry (0 = pure *extoni* and 1 = pure *pusillus* for sympatric individuals.

Supplementary Data 2

Description: List of individuals sequenced using ddRAD. The species is assigned only for allopatric individuals and provides the proportion of *pusillus* ancestry from fastSTRUCTURE for all individuals.
